# Supplementary material for: Protein coopted from a phage restriction system dictates orthogonal cell division plane selection in Staphylococcus aureus
Source: bioRxiv. 2023 Sep 3:2023.09.03.556088. Preprint. [Version 1] doi: 10.1101/2023.09.03.556088 (PMC10602043; doi:10.1101/2023.09.03.556088)
Supplement: 1 [file NIHPP2023.09.03.556088V1-supplement-1.pdf]

bioRxiv preprint doi: https://doi.org/10.1101/2023.09.03.556088; this version posted September 3, 2023. The copyright holder for this preprint (which was not certified by peer review) is the author/funder. This article is a US Government work. It is not subject to copyright under 17 USC 105 and is also made available for use under a CC0 license.

SUPPLEMENTAL DATA

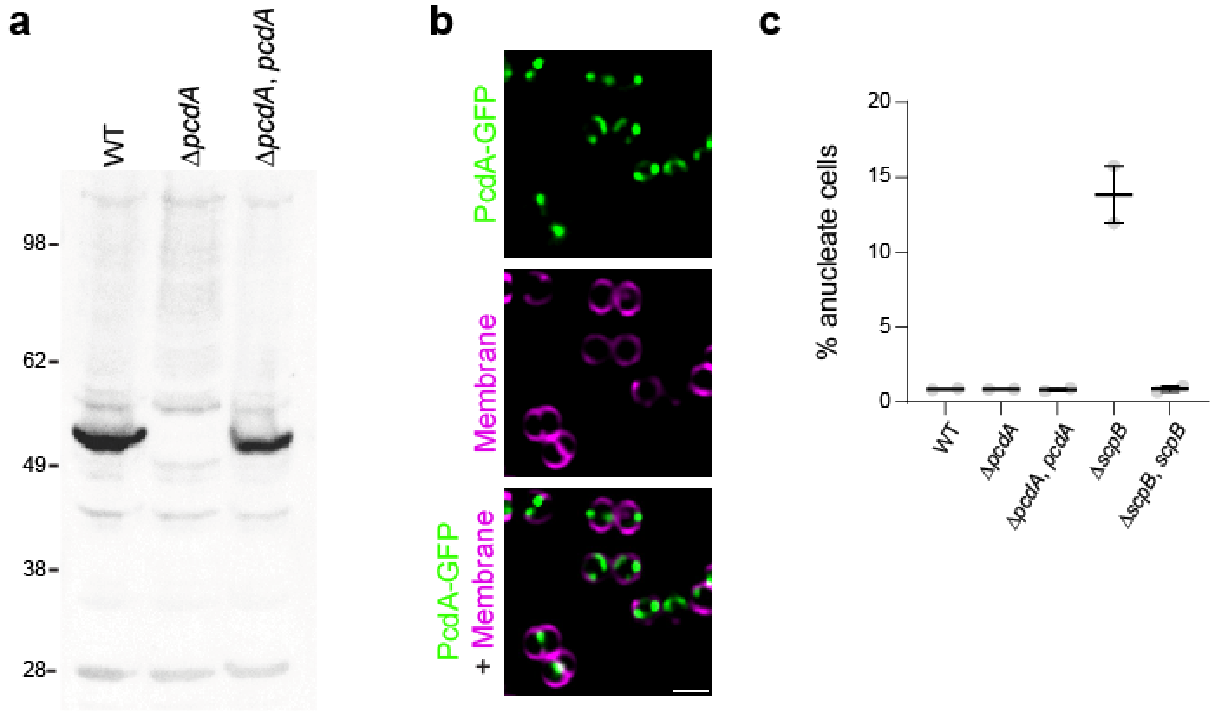

Figure S1. (a) Immunoblot using polyclonal antibodies against PcdA using extracts from WT, Δ*pcdA*, and Δ*pcdA* complemented at an ectopic chromosomal locus with *pcdA* strains. Predicted molecular weight for PcdA is ~53 kDa. Strains: JE2, FRL60, and FRL62. (b) Larger field of view showing subcellular localization of PcdA-sGFP in WT strain. First row: PcdA-sGFP (green); second row: membrane stained with FM4-64 (magenta); third row: overlay of PcdA-sGFP and membrane. Scale bar: 1 μm. Strain FRL28. (c) Graph showing percentage of anucleate cells for WT, Δ*pcdA*, complemented Δ*pcdA*, Δ*scpB*, and complemented Δ*scpB*. Data points are from two independent replicates where > 1,000 cells were analyzed for each strain; bars represent averages; errors: SEM. Strains: FRL60, FRL62, NE1085, and FRL12.

bioRxiv preprint doi: https://doi.org/10.1101/2023.09.03.556088; this version posted September 3, 2023. The copyright holder for this preprint (which was not certified by peer review) is the author/funder. This article is a US Government work. It is not subject to copyright under 17 USC 105 and is also made available for use under a CC0 license.

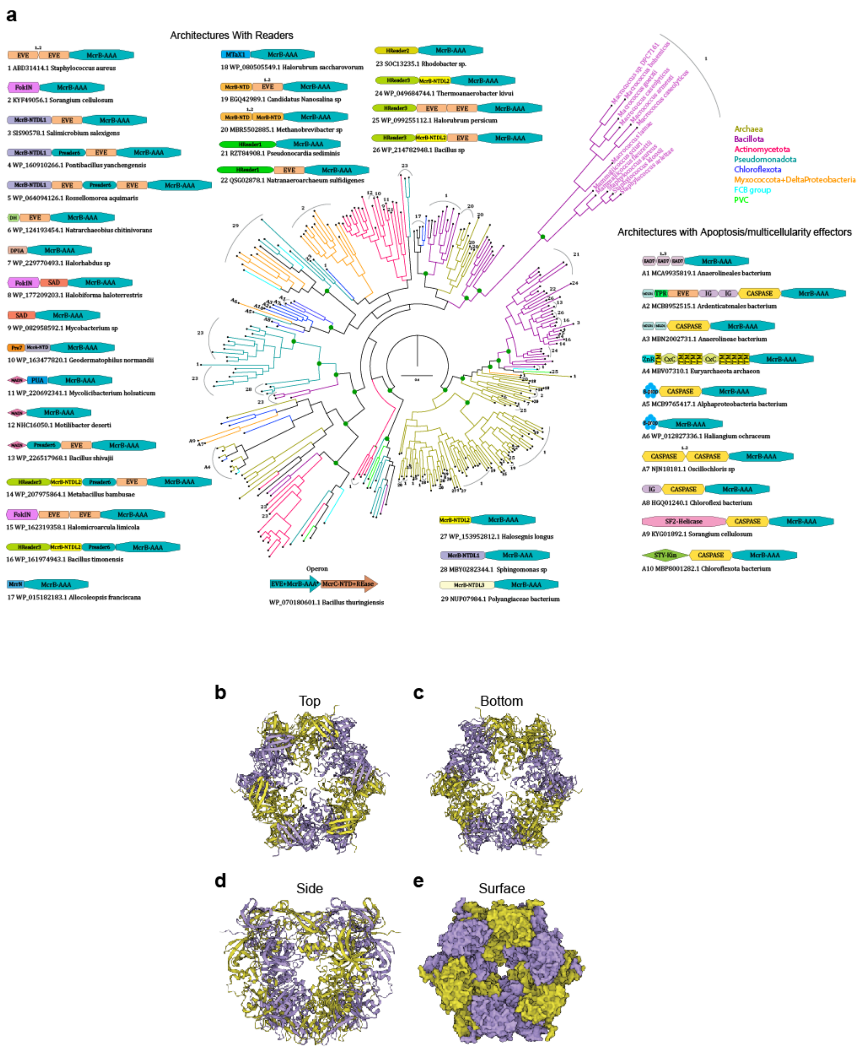

Figure S2. **Phylogenetic tree of McrB AAA+ ATPase from a Multiple Sequence Alignment with a curated set of proteins.** (a) The clades are colored by taxonomy as shown in the legend. Organism names are shown for the PcdA branch. Representative architectures and operon are shown with the accession and organism name below them. The arrows denote the genes in the operon with the "*" denoting the accession shown below it. The architectures are numbered, and the numbers are placed in the branches where they are found. Domains with variability in the number of tandem repeats are shown with a 1..2 or 1..3 above them. Abbreviations: FokIN – FokI-N-terminal-domain like, MAD-NTDL - MAD-NTD-like, DH - DpnI-HTH, DPUA - DCD-PUA, Pre7 - Prereader7, and MADN - MAD-NTD. The tree was generated using IQtree with the Dayhoff amino-acid exchange rate matrix which is empirically determined as one of the best fits. Key branches with boot strap support greater than 90% are shown with a green dot. (b-e) The predicted structure of a PcdA hexamer showing views along (b-c) the hexamer axis, (d) a side view perpendicular the axis, and (e) a surface view revealing a potential binding cavity. PcdA structures were generated using AF2.

bioRxiv preprint doi: https://doi.org/10.1101/2023.09.03.556088; this version posted September 3, 2023. The copyright holder for this preprint (which was not certified by peer review) is the author/funder. This article is a US Government work. It is not subject to copyright under 17 USC 105 and is also made available for use under a CC0 license.

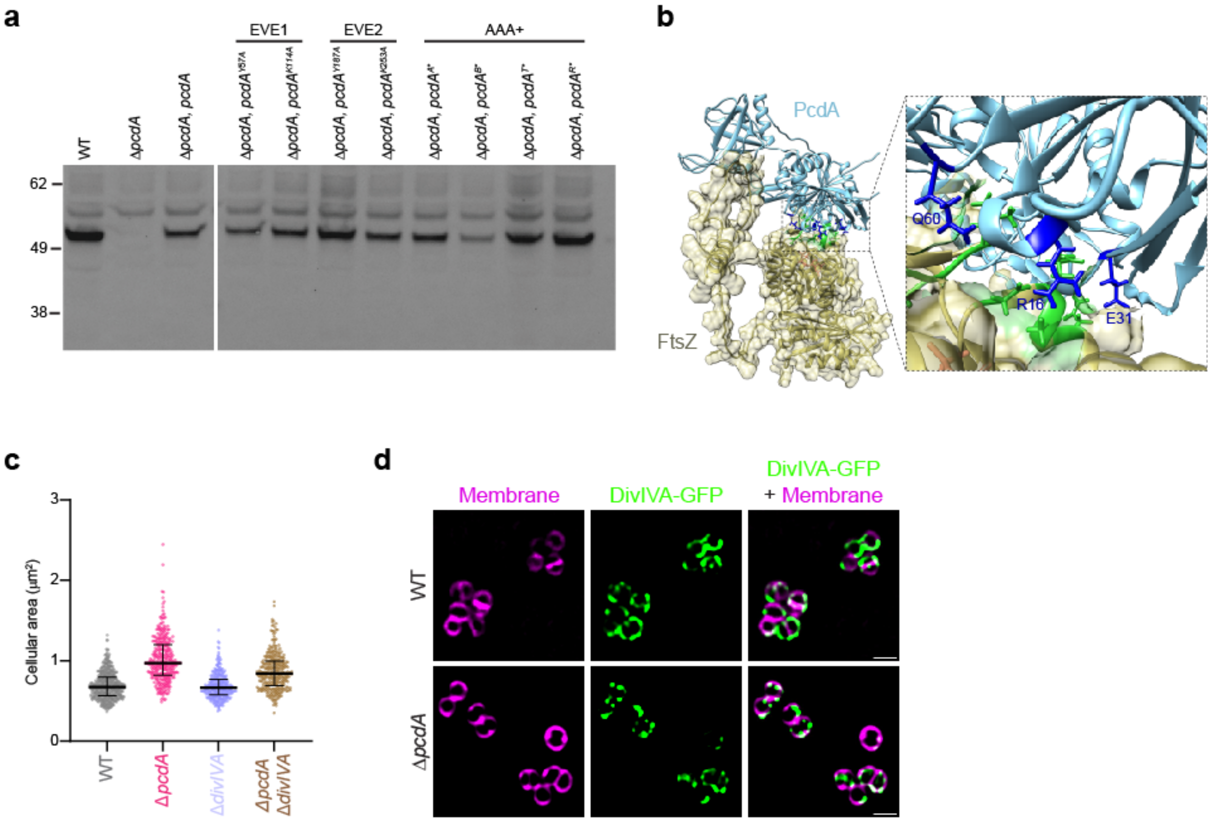

Figure S3. (a) Immunoblot using polyclonal antibodies against PcdA against cell extracts of WT, Δ*pcdA*, complemented Δ*pcdA*, or Δ*pcdA* complemented with indicated allele of *pcdA*. Strains: JE2, FRL60, FRL14, FRL34 – 41. (b) Protein complex model of a monomeric FtsZ (cyan) and PcdA (gold) predicted by AlphaFold-Multimer. A close-up section of the predicted interphase indicating residues R16, E31, and Q60 on PcdA may mediate the interaction with FtsZ.

(c) Cellular area (μm^2) of the indicated strains (n > 300 cells). Bars indicate median; whiskers indicate interquartile range. Strains: JE2, FRL60, FRL98, and FRL96. (d) Subcellular localization of DivIVA-sGFP in the WT and Δ*pcdA* strains. First column: membranes stained with FM4-64 (magenta); second column: DivIVA-sGFP (green); third column: overlay of membrane and DivIVA-sGFP. Scale bar: 1 μm. Strains: FRL113 and FRL114.

bioRxiv preprint doi: https://doi.org/10.1101/2023.09.03.556088; this version posted September 3, 2023. The copyright holder for this preprint (which was not certified by peer review) is the author/funder. This article is a US Government work. It is not subject to copyright under 17 USC 105 and is also made available for use under a CC0 license.

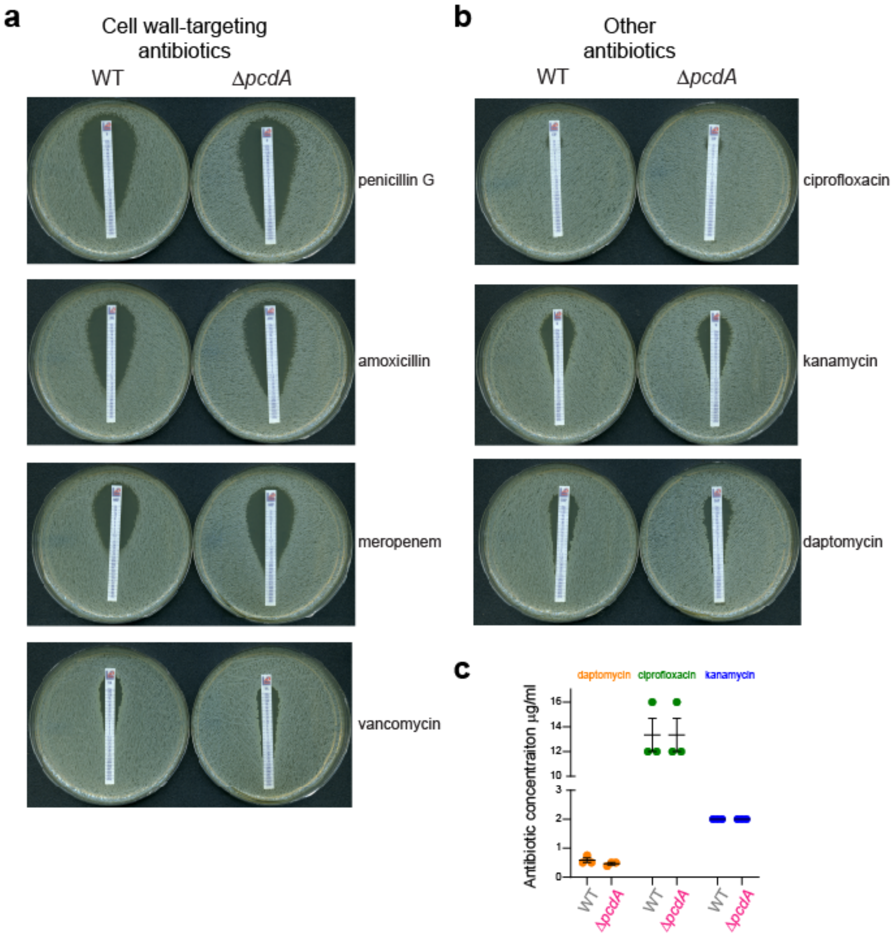

Figure S4. (a) Representative images of plates for MIC determination of cell wall-targeting antibiotics for the indicated strains. MIC was indicated by the intersection of the inhibition ellipse with the MIC strip. (b) Representative images of plates for MIC determination of antibiotics targeting other cellular process such as DNA metabolism (ciprofloxacin), protein synthesis (kanamycin), or cytoplasmic membrane (daptomycin). (c) MICs for JE2 wild type and Δ*pcdA* for the indicated antibiotics. Strains: JE2 and FRL60.
